# A quadratic trigonometric spline for curve modeling

**DOI:** 10.1371/journal.pone.0208015

**Published:** 2019-01-10

**Authors:** Shamaila Samreen, Muhammad Sarfraz, Malik Zawwar Hussain

**Affiliations:** 1 Department of Mathematics, University of Engineering and Technology, Lahore, Pakistan; 2 Department of Mathematics, University of the Punjab, Lahore, Pakistan; 3 Department of Information Science, College of Computing Sciences & Engineering, Kuwait University, Safat, Kuwait; Consiglio Nazionale delle Ricerche, ITALY

## Abstract

An imperative curve modeling technique has been established with a view to its applications in various disciplines of science, engineering and design. It is a new spline method using piecewise quadratic trigonometric functions. It possesses error bounds of order 3. The proposed curve model also owns the most favorable geometric properties. The proposed spline method accomplishes *C*^2^ smoothness and produces a Quadratic Trigonometric Spline (QTS) with the view to its applications in curve design and control. It produces a *C*^2^ quadratic trigonometric alternative to the traditional cubic polynomial spline (CPS) because of having four control points in its piecewise description. The comparison analysis of QTS and CPS verifies the QTS as better alternate to CPS. Also, the time analysis proves QTS computationally efficient than CPS.

## 2. Introduction

Designing curves, especially robust curves, which are controllable, well behaved and easily worked out, contributes a special role in computer graphics and geometric modeling. A number of applications of these vigorous curves in modeling objects, CAD/CAM, font designing, object recognition, medical imaging and fingerprints recognition are the motivations in the direction of curve designing. Besides the applications, the significance of curve designing in computer visualization, robotics and even in broad casting is the inspiration to do research. In the existing literature, a wide-ranging effort has been put in the course of curve modeling. For reference, the readers are directed to [1–25].

It is favorable to adopt an effective, robust, well controlled and visually nice method as a key of many problems on a single platform. In the area of geometric modeling and computer graphics, a cubic polynomial spline (CPS) curve method is supposed to be an adequate approach in the current literature [1–25]. Deliberation of a stable and faster new spline, as a better alternate to CPS, accompanied by piecewise descriptions can be a better proposal for this paper. Thus, providing a quadratic trigonometric spline alternate of a CPS, which not only keeps decent attributes of CPS but is also computationally less expensive, could be a better choice for designers and engineers.

It is worthy to present a method for a class of trigonometric splines with a support of ideal geometric properties and efficiency. Boehm [1] constructed the curvature continuous curves and surfaces. Barsky [2] developed B-splines basis to Beta splines. He preserved the geometric smoothness property of the curve modeling while enabling the conditions of continuity on the splines at different knots to be assorted by certain shape parameters; thus, providing more flexibility. Cline [3] used the splines under tension for the curve fitting. Dierckx and Tytgat [4], presented a proficient procedure to compute the Bézier points of a generalized cubic *β*-spline curve and showed the connection with multiple knot insertion. They also determined the *β*-spline vertices for a composite *G*^2^ Bézier curve. Farin [5] has the detail study on curves and surfaces for Computer-Aided Geometric Design (CAGD). Foley [6] constructed the weighted spline to acquire the tension locally. Foley [7] built B-spline like local basis functions for weighted nu-splines. The local basis for *GC*^2^ splines was introduced by Lewis [8]. Nielson [9–10] described the classification of splines with tension and offered the alternates of splines under tension. The developed splines were the generalization of CPS, nu-splines and weighted splines. A general setting was specified by Pruess [11], for smooth interpolation of the splines depending on a parameter. Sarfraz [12–17] constructed various spline techniques for shape modeling using various spline which are a big contribution in literature. Sarfraz [18] built a spline for object designing. The developed spline presents the generalization of Ball’s cubic spline. Hussain et al. [19] developed the shape preserving method using rational quadratic trigonometric function. Later Sarfraz et al. [20–23] and Samreen et al. [24] constructed different spline methods for shape designing using a quadratic trigonometric splines with well controlled shape influences of parameters.

This paper presents a quadratic trigonometric spline (QTS) as a better alternate to the CPS. The proposed QTS method is robust, geometrically ideal, and computationally faster. The paper has been organized in various sections. Section 3 offers the QTS and CPS whereas Section 4 calculates the error analysis of QTS using Peano-Kernel Theorem. Section 5 proposes the new *C*^2^ QTS, it is presented here in interpolation form. An efficient and compact algorithm, to compute and plot the curve design, is also part of this section. Geometric properties of the proposed QTS are logically described and proved in Section 6. These properties include partition of unity, positivity, end-point interpolation, convex hull, affine invariance and variation diminishing. Section 7 provides a comparison analysis of the proposed QTS versus CPS. In this regard, area between two spline curves, visual differences, time elapsed of two splines and error analysis of both splines are taken into account. Section 8 is dedicated to specify the advantageous features of the proposed QTS method and analyzes the superiority of QTS over CPS. Finally, Section 9 concludes the paper.

## 3. The proposed spline

To describe the QTS, assume the data points $F_{i}\epsilon R^{n}$ at the knots *t*_*i*_, *i* = 0,1,…,*n*−1, where *t*_0_<*t*_1_<,…,<*t*_*n*_. For *tϵ*[*t*_*i*_,*t*_*i*+1_], *i* = 0,1,…,*n*−1, let $V_{i},W_{i}\epsilon R^{n}$ and

$$P_{i}(t)={\left(1-Sin\theta \right)}^{2}F_{i}+2(1-Sin\theta )Sin\theta V_{i}+2(1-Cos\theta )Cos\theta W_{i}+(1-Cos{\theta )}^{2}F_{i+1}$$ (1)

where, $\theta =\theta (t)=(\frac{t-t_{i}}{h_{i}})\frac{\pi }{2},\;h_{i}=t_{i+1}-t_{i},0\leq \theta \leq \frac{\pi }{2}$, and

$$V_{i}=F_{i}+\frac{h_{i}}{\pi }D_{i},\;W_{i}=F_{i+1}-\frac{h_{i}}{\pi }D_{i+1}.$$ (2)

Also,

$${P(t}_{i})=F_{i},\;{P^{\prime }(t}_{i})=D_{i},{P^{\prime }(t}_{i+1})=D_{i+1},{P(t}_{i+1})=F_{i+1}.$$ (3)

Then, eventually piecewise QTS behaves like a Hermite CPS. The Hermite CPS, defined for *tϵ*[*t*_*i*_,*t*_*i*+1_],*t*_*i*_<*t*_*i*+1_,∀ *i* = 0,1,…,*n*−1, with the data points *F*_*i*_ and *V*_*i*_, $W_{i}\epsilon R^{n}$, is described by:

$$P_{i}(t)={\left(1-\theta \right)}^{3}F_{i}+{\left(1-\theta \right)}^{2}\theta V_{i}+(1-\theta )\theta^{2}W_{i}+\theta^{3}F_{i+1},$$ (4)

where, $\theta =\theta (t)=(\frac{t-t_{i}}{h_{i}}),\;h_{i}=t_{i+1}-t_{i},\;0\leq \theta \leq 1$, and

$$V_{i}=F_{i}+\frac{h_{i}}{3}D_{i},\;W_{i}=F_{i+1}-\frac{h_{i}}{3}D_{i+1}.$$ (5)

Also, it can be obviously seen, the CPS fulfills the interpolation properties as in (3).

## 4. Error analysis of QTS

The error of QTS function (1) is calculated in this segment while the function *F*(*t*)∈[*t*_0_,*t*_*n*_] is interpolated. Since the quadratic trigonometric function interpolated locally without losing generality, the error is calculated in the subinterval [*t*_*i*_,*t*_*i*+1_]. Let *P*(*t*) be the quadratic trigonometric function of *F*(*t*) interpolated in [*t*_*i*_,*t*_*i*+1_] as defined in (1) then by applying the Peano-Kernel Theorem, $E[f]=F(t)-P(t)=\frac{1}{2}\int_{t_{i}}^{t_{i+1}}F^{3}(\tau )E_{t}[{\left(t-\tau \right)}_{+}^{2}]d\tau$ where $E_{t}[{\left(t-\tau \right)}_{+}^{2}]$ is the kernel of integral defined for the quadratic trigonometric function given by

$$E_{t}[{\left(t-\tau \right)}_{+}^{2}]=\left\{ \begin{array}{l} u_{1}(\tau ,t),\;t_{i}<\tau <t,\\ v_{1}(\tau ,t),\;t<\tau <t_{i+1}, \end{array}\right.$$ (6)

with, $u_{1}(\tau ,t)={\left(t-\tau \right)}^{2}-\{2cos\theta (1-cos\theta )[{\left(t_{i+1}-\tau \right)}^{2}-\frac{2h_{i}}{\pi }(t_{i+1}-\tau )]+{\left(1-cos\theta \right)}^{2}{\left(t_{i+1}-\tau \right)}^{2}\}$ and $v_{1}(\tau ,t)=-\{2cos\theta (1-cos\theta )[{\left(t_{i+1}-\tau \right)}^{2}-\frac{2h_{i}}{\pi }(t_{i+1}-\tau )]+{\left(1-cos\theta \right)}^{2}{\left(t_{i+1}-\tau \right)}^{2}\}.$

The proof of the error estimation mainly consisting of two steps; discussing the properties of function *u*_1_(*τ*,*t*) and *v*_1_(*τ*,*t*) and in the second step, computing the absolute values $\int_{t_{i}}^{t}|u_{1}(\tau ,t)|d\tau$ and $\int_{t}^{t_{i+1}}|v_{1}(\tau ,t)|d\tau .$

Step 1: *u*_1_(*τ*,*t*) and *v*_1_(*τ*,*t*),*τ*∈[*t*_*i*_,*t*], are the quadratic trigonometric functions of *τ*. Also for all $\theta \in [0,\frac{\pi }{2}]$, *u*(*t*_*i*_,*t*) = 0 and *v*(*t*_*i*+1_,*t*) = 0.

Now to calculate the roots of *u*_1_(*τ*,*t*) and *v*_1_(*τ*,*t*), put *τ* = *t* in Eq (6) as $u_{1}(t,t)={\left(t-t\right)}^{2}-\{2cos\theta (1-cos\theta )[{\left(t_{i+1}-t\right)}^{2}-\frac{2h_{i}}{\pi }(t_{i+1}-t)]+{\left(1-cos\theta \right)}^{2}{\left(t_{i+1}-t\right)}^{2}\}$ and $v_{1}(t,t)=-\{2cos\theta (1-cos\theta )[{\left(t_{i+1}-t\right)}^{2}-\frac{2h_{i}}{\pi }(t_{i+1}-t)]+{\left(1-cos\theta \right)}^{2}{\left(t_{i+1}-t\right)}^{2}\},$ where, $t_{i+1}-t=t_{i+1}-t_{i}+t_{i}-t=h_{i}(1-\frac{2\theta }{\pi })=h_{i}(1-\sigma ),$ with $\sigma =\frac{2\theta }{\pi },$ implies that $u_{1}(t,t)=-\{2cos\theta (1-cos\theta )[{h_{i}^{2}(1-\sigma )}^{2}-\frac{2h_{i}^{2}}{\pi }(1-\sigma )]+{\left(1-cos\theta \right)}^{2}{h_{i}^{2}(1-\sigma )}^{2}\}=v_{1}(t,t).$

Let, $(1-\sigma )-\frac{2}{\pi }=0,$ be an equation defined in *θ*, its root in $\left(0,\frac{\pi }{2}\right)$ is

$$\sigma^{*}=1-\frac{2}{\pi },$$ (7)

It implies, $\theta^{*}=\frac{\pi }{2}-1.$ It can easily be observed that, for *σ*≤σ*,*u*_1_(*t*,*t*)≤0 and for *σ*≥σ*,*u*_1_(*t*,*t*)≥0. Consider (*t*_*i*+1_−*τ*) = (*t*_*i*+1_−*t*+*t*−*τ*) = *h*_*i*_(1−*σ*)+(*t*−*τ*). Now rewrite *u*_1_(*τ*,*t*) to observed sign of *u*_1_(*τ*,*t*) in [*t*_*i*_,*t*], as $u_{1}(\tau ,t)={\left(t-\tau \right)}^{2}(1-A_{2}-A_{3})+(t-\tau )2h_{i}\{\frac{A_{2}}{\pi }-(1-\sigma ){(A}_{2}+A_{3})\}+(1-\sigma )h_{i}^{2}\{\frac{{2A}_{2}}{\pi }-(1-\sigma ){(A}_{2}+A_{3})\}.$

Then the two roots of *u*_1_(*τ*,*t*) are $\tau_{1}=\frac{h_{i}(B+D)}{A}+t$ and $\tau_{2}=\frac{h_{i}(B-D)}{A}+t,$ where $A=1-A_{2}-A_{3},\;B=\frac{A_{2}}{\pi }-(1-\sigma ){(A}_{2}+A_{3})$ and $D=\sqrt{{\left(\frac{A_{2}}{\pi }-(1-\sigma ){(A}_{2}+A_{3})\right)}^{2}-(1-A_{2}-A_{3})(1-\sigma )(\frac{{2A}_{2}}{\pi }-(1-\sigma ){(A}_{2}+A_{3}))}$ with the real values.

Similarly, it can be noted that for *σ*≤σ*,*v*_1_(*t*,*t*)≤0 and for *σ*≥σ*,*v*_1_(*t*,*t*)≥0. To show this sign change behavior rewrite *v*_1_(*τ*,*t*) as $v_{1}(\tau ,t)={\left(t-\tau \right)}^{2}(-A_{2}-A_{3})+(t-\tau )2h_{i}\{\frac{A_{2}}{\pi }-(1-\sigma ){(A}_{2}+A_{3})\}+(1-\sigma )h_{i}^{2}\{\frac{{2A}_{2}}{\pi }-(1-\sigma ){(A}_{2}+A_{3})\},$ Then the two roots of *v*_1_(*τ*,*t*) are $\tau_{1}^{*}=t_{i+1}$ and $\tau_{2}^{*}=t_{i+1}-\frac{2h_{i}A_{2}}{\pi (A_{2}+A_{3})},$ using (*t*_*i*+1_−*τ*) = (*t*_*i*+1_−*t*+*t*−*τ*) = *h*_*i*_(1−*σ*)+(*t*−*τ*).

Step 2: While *σ*>σ*, *u*_1_(*τ*,*t*)>0 and when *σ*<σ*, *u*_1_(*τ*,*t*)<0. It can also noted that *t*_*i*_<*τ*_2_<*τ*_1_<*t*. Thus, for *σ*<σ*,*u*_1_(*τ*,*t*)<0 ∀ *τ*∈[*t*_*i*_,*t*],

$$\int_{t_{i}}^{t}|u_{1}(\tau ,t)|d\tau =\int_{t_{i}}^{t}(-u_{1}(\tau ,t))d\tau =-\frac{1}{3}(1-A_{2}-A_{3})h_{i}^{3}\sigma^{3}-(\frac{A_{2}}{\pi }-(1-\sigma ){(A}_{2}+A_{3}))h_{i}^{3}\sigma^{2}-(1-\sigma )h_{i}^{3}\sigma \{\frac{{2A}_{2}}{\pi }-(1-\sigma ){(A}_{2}+A_{3})\}.$$ (8)

Also for *σ*>σ*, *u*_1_.(*τ*,*t*) changes sign on both sides of *τ*_1_ and *τ*_2_, so

$$\int_{t_{i}}^{t}|u_{1}(\tau ,t)|d\tau =\int_{t_{i}}^{\tau_{2}}u_{1}(\tau ,t)d\tau +\int_{\tau_{2}}^{\tau_{1}}(-u_{1}(\tau ,t))d\tau +\int_{\tau_{1}}^{t}u_{1}(\tau ,t)d\tau =\frac{1}{3}(1-A_{2}-A_{3})[-h_{i}^{3}\sigma^{3}+2h_{i}^{3}{\left(\frac{B-D}{A}\right)}^{3}-2h_{i}^{3}{\left(\frac{B+D}{A}\right)}^{3}]+h_{i}\{\frac{A_{2}}{\pi }-(1-\sigma ){(A}_{2}+A_{3})\}[h_{i}^{2}\sigma^{2}-2h_{i}^{2}{\left(\frac{B-D}{A}\right)}^{2}+2h_{i}^{2}{\left(\frac{B+D}{A}\right)}^{2}]+(1-\sigma )h_{i}^{2}\{\frac{{2A}_{2}}{\pi }-(1-\sigma ){(A}_{2}+A_{3})\}[h_{i}\sigma +{2h}_{i}(\frac{B-D}{A})-2h_{i}(\frac{B+D}{A})].$$ (9)

Similarly, it can display easily that when *σ*≤σ*, *v*_1_(*τ*,*t*) moves from negative side to positive side of *τ*_1*_, and while *σ*≥σ*, *v*_1_(*τ*,*t*)>0 in (*t*,*t*_*i*+1_), where σ* is given by Eq (7). Thus while taking *σ*≤σ*

$$\int_{t}^{t_{i+1}}|v_{1}(\tau ,t)|d\tau =\int_{t}^{\tau_{2}^{*}}(-v_{1}(\tau ,t))d\tau +\int_{\tau_{2}^{*}}^{t_{i+1}}v_{1}(\tau ,t)d\tau =(-\frac{1}{3}{(A}_{2}+A_{3}))\left[2{{(h}_{i}(\sigma -1)+\frac{2h_{i}A_{2}}{\pi {(A}_{2}+A_{3})})}^{3}-h_{i}^{3}{\left(\sigma -1\right)}^{3}\right]+h_{i}\{\frac{A_{2}}{\pi }-(1-\sigma ){(A}_{2}+A_{3})\}\left[2{{(h}_{i}(\sigma -1)+\frac{2h_{i}A_{2}}{\pi {(A}_{2}+A_{3})})}^{2}-h_{i}^{2}{\left(\sigma -1\right)}^{2}\right]+(1-\sigma )h_{i}^{2}\{\frac{{2A}_{2}}{\pi }-(1-\sigma ){(A}_{2}+A_{3})\}[{(h}_{i}(\sigma -1)+\frac{4h_{i}A_{2}}{\pi {(A}_{2}+A_{3})}],$$ (10)

but while *σ*≥σ*,

$$\int_{t}^{t_{i+1}}|v_{1}(\tau ,t)|d\tau =\int_{t}^{t_{i+1}}v_{1}(\tau ,t)d\tau =-\frac{1}{3}{(A}_{2}+A_{3})h_{i}^{3}{\left(1-\sigma \right)}^{3}-h_{i}\{\frac{A_{2}}{\pi }-(1-\sigma ){(A}_{2}+A_{3})\}h_{i}^{2}{\left(1-\sigma \right)}^{2}+(1-\sigma )h_{i}^{2}\{\frac{{2A}_{2}}{\pi }-(1-\sigma ){(A}_{2}+A_{3})\}h_{i}(1-\sigma ).$$ (11)

So, combining (8) and (10), the following result holds for *σ*≤σ*, $|F(t)-P(t)|\leq \frac{\left\|F^{\left(3\right)}\right\|}{2}\int_{t_{i}}^{t_{i+1}}|R_{t}[{\left(t-\tau \right)}_{+}^{2}]|d\tau =\|F^{\left(3\right)}\|h_{i}^{3}\gamma^{*}(\sigma ),$ where γ*(*σ*) is defined by

$$\gamma^{*}(\sigma )=\int_{t_{i}}^{t}(-u_{1}(\tau ,t))d\tau +\int_{t}^{\tau_{2}^{*}}(-v_{1}(\tau ,t))d\tau +\int_{\tau_{2}^{*}}^{t_{i+1}}v_{1}(\tau ,t)d\tau =-\frac{1}{3}(1-A_{2}-A_{3})h_{i}^{3}\sigma^{3}-(\frac{A_{2}}{\pi }-(1-\sigma ){(A}_{2}+A_{3}))h_{i}^{3}\sigma^{2}-(1-\sigma )h_{i}^{3}\sigma \{\frac{{2A}_{2}}{\pi }-(1-\sigma ){(A}_{2}+A_{3})\}+\left(-\frac{1}{3}{(A}_{2}+A_{3})\right)\left[2{{(h}_{i}(\sigma -1)+\frac{2h_{i}A_{2}}{\pi {(A}_{2}+A_{3})})}^{3}-h_{i}^{3}{\left(\sigma -1\right)}^{3}\right]+h_{i}\{\frac{A_{2}}{\pi }-(1-\sigma ){(A}_{2}+A_{3})\}\left[2{{(h}_{i}(\sigma -1)+\frac{2h_{i}A_{2}}{\pi {(A}_{2}+A_{3})})}^{2}-h_{i}^{2}{\left(\sigma -1\right)}^{2}\right]+(1-\sigma )h_{i}^{2}\{\frac{{2A}_{2}}{\pi }-(1-\sigma ){(A}_{2}+A_{3})\}[{(h}_{i}(\sigma -1)+\frac{4h_{i}A_{2}}{\pi {(A}_{2}+A_{3})}],$$ (12)

and the result obtained for *σ*≥σ^*^, by combining (9) and (11), $|F(t)-P(t)|\leq \frac{\left\|F^{\left(3\right)}\right\|}{2}\int_{t_{i}}^{t_{i+1}}|R_{t}[{\left(t-\tau \right)}_{+}^{2}]|d\tau =\|F^{\left(3\right)}\|h_{i}^{3}\gamma_{*}(\sigma ),$ where,

$$\gamma_{*}(\sigma )=\int_{t_{i}}^{\tau_{2}}u_{1}(\tau ,t)d\tau +\int_{\tau_{2}}^{\tau_{1}}(-u_{1}(\tau ,t))d\tau +\int_{\tau_{1}}^{t}u_{1}(\tau ,t)d\tau +\int_{t}^{t_{i+1}}v_{1}(\tau ,t)d\tau =\frac{1}{3}(1-A_{2}-A_{3})[-h_{i}^{3}\sigma^{3}+2h_{i}^{3}{\left(\frac{B-D}{A}\right)}^{3}-2h_{i}^{3}{\left(\frac{B+D}{A}\right)}^{3}]+h_{i}\{\frac{A_{2}}{\pi }-(1-\sigma ){(A}_{2}+A_{3})\}[h_{i}^{2}\sigma^{2}-2h_{i}^{2}{\left(\frac{B-D}{A}\right)}^{2}+2h_{i}^{2}{\left(\frac{B+D}{A}\right)}^{2}]+(1-\sigma )h_{i}^{2}\{\frac{{2A}_{2}}{\pi }-(1-\sigma ){(A}_{2}+A_{3})\}[h_{i}\sigma +{2h}_{i}(\frac{B-D}{A})-2h_{i}(\frac{B+D}{A})]-\frac{1}{3}{(A}_{2}+A_{3})h_{i}^{3}{\left(1-\sigma \right)}^{3}-h_{i}\{\frac{A_{2}}{\pi }-(1-\sigma ){(A}_{2}+A_{3})\}h_{i}^{2}{\left(1-\sigma \right)}^{2}+(1-\sigma )h_{i}^{2}\{\frac{{2A}_{2}}{\pi }-(1-\sigma ){(A}_{2}+A_{3})\}h_{i}(1-\sigma ).$$ (13)

The above analysis yields the following results.

**Theorem 1.** Let *P*(*t*) be be the quadratic trigonometric function, as defined in (1). The error estimation of *P*(*t*) holds the followings:

$$|F(t)-P(t)|\leq \|F^{\left(3\right)}\|h_{i}^{3}d^{*},\;where,\;d^{*}=\max_{0\leq \theta \leq 1}\gamma (\sigma ),\;and\;\gamma (\theta )=\left\{ \begin{array}{c} \gamma^{*}(\sigma )\;0\leq \sigma \leq \sigma^{*},\\ \gamma_{*}(\sigma )\;\sigma_{*}\leq \sigma \leq 1, \end{array}\right.$$

where γ*(*σ*) and γ_*_(*σ*) are respectively, taken from (12) and (13).

**Remark 1.** The coefficient of error term, d* can be defined as:

$$d^{*}=\max\{\max_{0\leq \theta \leq 1/2}\gamma^{*}(\sigma ),\max_{1/2\leq \theta \leq 1}\gamma_{*}(\sigma )\}.$$ (14)

## 5. Formation of *C*^2^ QTS

As seen in Section 2, the QTS is *C*^1^ by its construction. A *C*^2^ QTS can be formed by applying *C*^2^ continuity at the joints of curve segments as follows:

$${P_{i}^{\prime \prime }(t}_{i})={P_{i-1}^{\prime \prime }(t}_{i}).$$ (15)

From the second order derivative of (1), we simply achieve the followings:

$${P_{i}^{\prime \prime }(t}_{i})=\frac{\pi^{2}}{2h_{i}^{2}}F_{i}-\frac{\pi^{2}}{h_{i}^{2}}V_{i}+\frac{\pi^{2}}{2h_{i}^{2}}W_{i},$$ (16)

and

$${P_{i-1}^{\prime \prime }(t}_{i})=\frac{\pi^{2}}{2h_{i}^{2}}F_{i}+\frac{\pi^{2}}{{2h}_{i}^{2}}V_{i-1}-\frac{\pi^{2}}{h_{i}^{2}}W_{i-1}.$$ (17)

Let

$$\Delta_{i}=\frac{F_{i+1}-F_{i}}{h_{i}},$$ (18)

then, the Eqs (16) and (17), respectively, crop to:

$${P_{i}^{\prime \prime }(t}_{i})=\frac{\pi }{2h_{i}}(\Delta_{i}\pi -2D_{i}-D_{i+1}),$$ (19)

and

$${P_{i-1}^{\prime \prime }(t}_{i})=\frac{\pi }{2h_{i-1}}(-\Delta_{i-1}\pi +D_{i-1}+{2D}_{i}).$$ (20)

Also

$$P_{i-1}^{\prime }(t_{i})=D_{i}=P_{i}^{\prime }(t_{i}),$$ (21)

Thus, using (19), (20) and (21) in (14), a tri-diagonal system of consistency equations is acquired by the followings:

$$\frac{1}{{2h}_{i-1}}D_{i-1}+\left(\frac{1}{h_{i-1}}+\frac{1}{h_{i}}\right)D_{i}+\frac{1}{{2h}_{i}}D_{i+1}=\frac{\pi }{{2h}_{i}}\Delta_{i}+\frac{\pi }{2h_{i-1}}\Delta_{i-1}.$$ (22)

The above system can be expressed by the following matrix:

$$[\begin{array}{ccc} \frac{1}{h_{1}}+\frac{1}{h_{0}} & \frac{1}{2h_{1}} & 0\\ \frac{1}{2h_{1}} & \frac{1}{h_{2}}+\frac{1}{h_{1}} & \frac{1}{2h_{1}}\\ \ddots & \ddots & \ddots \\ \;0 & \frac{1}{2h_{n-2}} & \frac{1}{h_{n-1}}+\frac{1}{h_{n-2}} \end{array}][\begin{array}{c} D_{1}\\ D2\\ \vdots \\ D_{n-1} \end{array}]=$$ (23)

It is supposed that end conditions *D*_0_ and *D*_*n*_ are given. One can choose the arbitrary end conditions by choice. Overall, for open curves, the end conditions *D*_0_ and *D*_*n*_ are determined using the given data points as follows:

$$D_{0}=2(F_{1}-F_{0})-\frac{F_{2}-F_{0}}{2},$$ (24)

$$D_{n}=2(F_{n}-F_{n-1})-\frac{F_{n}-F_{n-2}}{2}.$$ (25)

For closed curves, the following periodic end conditions *D*_0_ = *D*_*n*_ are assumed:

$$F_{n}=F_{0},F_{n-1}=F_{-1},F_{n+1}=F_{1}.$$ (26)

Thus, for the appropriate end conditions, the above system (22) is a tri-diagonal linear system and is diagonally dominant. It also retains unique solutions for *Di*′*s* and hence a unique solution for QTS. To solve the tri-diagonal system for *Di*′*s*, it is efficient to accomplish the LU-decomposition method. Thus, the above conversation can be concise as follows:

**Theorem 2.** The *C*^2^ QTS exists and has a unique solution.

### 5.1. Algorithm design

The above discussion is summarized in some steps here. It is done by designing a suitable algorithm for the curve modeling applications. In this way, a user may have an open choice to play with a curve at its own satisfaction level. It can be achieved by an appropriate algorithm design as follows:

Step 1:Input control points *F*_*i*_’s.
Step 2:Compute the tangents *D*_*i*_’s, from the data points in Step 1, using system of Eqs (23–26).
Step 3:Compute the QTS curve (1) of Section 3.

This is a simple algorithm to implement QTS. The algorithm for CPS is also similar and hence not been mentioned here. Both of CPS and QTS have been implemented using Matlab [25] software.

### 5.2. Data of objects

The data of various objects drawn in the Figs is given in Table 1.

**Table 1:** Table of data of objects.

#	Different Objects.	Data of the objects
(1)	Circle	*x*	0 1 2 1
		*y*	1 0 1 2
(2)	Lamp	*x*	0 0 .5 .2 .2 .5 -.2 -2 -.2 1.7 3.5 1.7 1 1.3 1.3 1 1.5 1.5
		*y*	0 .5 1 1.5 2 2.5 3 3 5 5 3 3 2.5 2 1.5 1 .5 0
(3)	Car			*x*	.5 1.4 1.7 2.4 2.9 4.6 5 5.5 5.9 7 7.3 6.4 5.5 2.9 2.3 0.5
*y*	.5 .5 0.9 0.9 .5 .5 .9 .9 .5 .5 2 2 2.5 2.5 1.8 1.5
		(4)	Flower	*x*	0 1 2 1 2 1 0 1
*y*	0 1 0 1 2 1 2 1
		(5)	Guitar	*x*	1.5 3 4 5.5 6 5 5.5 5 4 4 4 3 3 3 2 1.5 2 1
*y*	1 .5 .5 1 3 4 5 6.2 7 9 12 12 9 7 6.2 5 4 3
		(6)	Vase	*x*	2.5 1 2.3 2.3 0 1 4 5 2.7 2.7 4
*y*	4 3.5 3.5 2.5 1.5 0 0 1.5 2.5 3.5 3.5
		(7)	‘A’ alphabet	*x*	0 1 1.2 1.8 2 3 1.5
*y*	0 0 1 1 0 0 5.5

**Fig 1:**
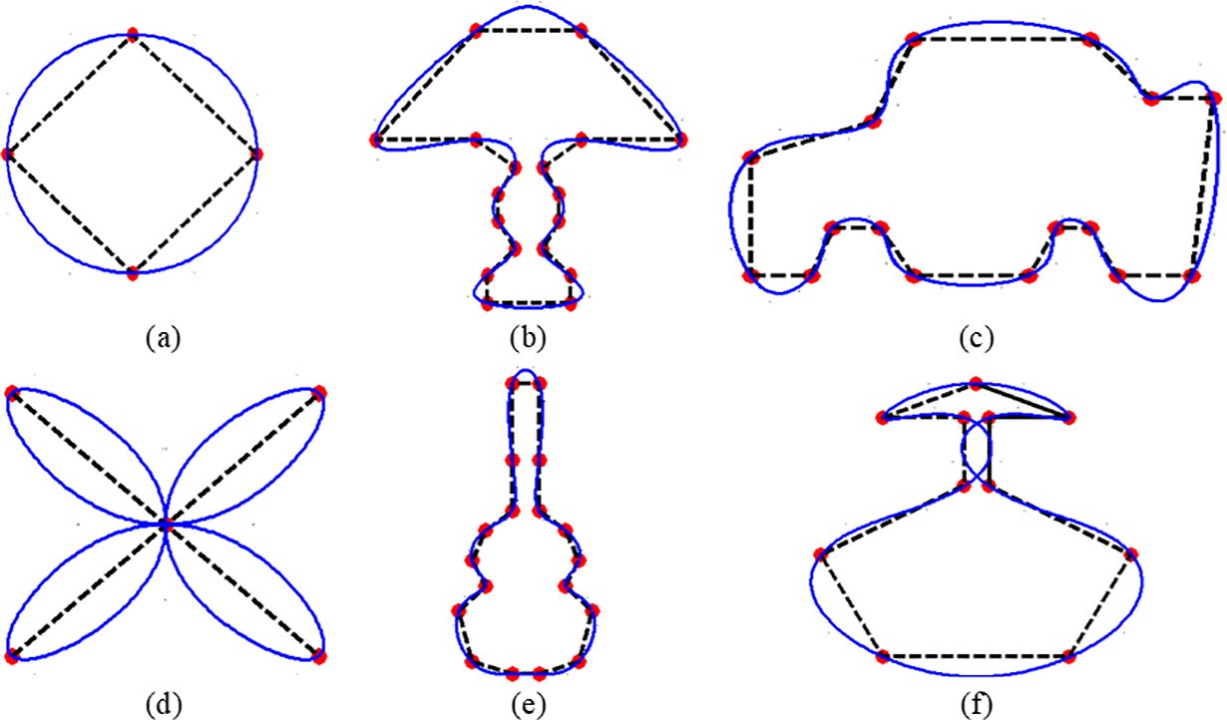
The data points of different objects (a) Circle, (b) Lamp, (c) Car, (d) Flower, (e) Guitar, (f) Vase, are interpolated by QTS with periodic end conditions.

For the chosen data points $F_{i}\in R^{2},i=1,\ldots ,n,$ *C*^2^ QTSs are demonstrated in Fig 1A–1F.

## 6. Geometric properties of QTS

The QTS owns several ideal geometric properties discussed by the propositions as follow:

**Proposition 1 (Convex Hull Property (CHP)):** The QTS curve lies completely inside the convex hull determined by its control polygon.

**Fig 2:**
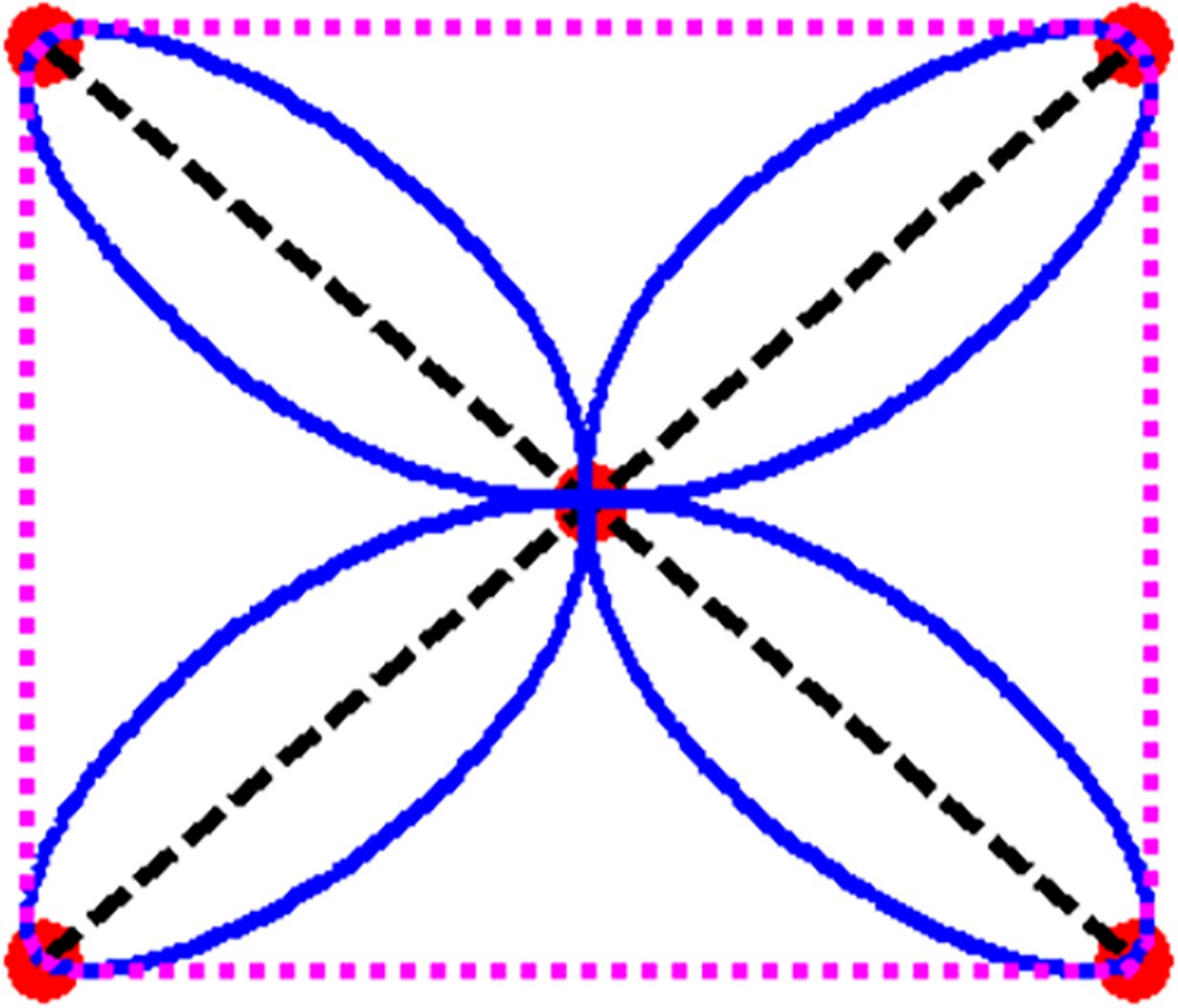
CHP using Flower data and its QTS.

**Proof:** By re-writing Eq (1) as:

$$P_{i}(t)=B_{0}(\theta )F_{i}+B_{1}(\theta )V_{i}+B_{2}(\theta )W_{i}+B_{3}(\theta )F_{i+1},$$ (27)

where *B*_*j*_(*θ*)≥0,*j* = 0,…,3, are Bernstein Bézier like functions and $\sum_{j=0}^{3}B_{j}(\theta )=1$. Therefore, the QTS curve lies within the convex hull as shown in Fig 2.

**Proposition 2 (Affine Invariance Property (AIP)):** Let $P(t)=\sum_{j=0}^{3}d_{j}B_{j}(t)$ be the *C*^2^ QTS curve and $d_{j}=\{F_{i}{,V}_{i},W_{i},F_{i+1}\}\in R^{n}$ be the control points. Then *C*^2^ QTS curve is invariant under affine transformation.

**Proof:** Let, (*x*_1_,*y*_1_) = (*a*_1_*x*+*a*_2_*y*+*a*_3_,*b*_1_*x*+*b*_2_*y*+*b*_3_), be an affine transformation and *d*_*j*_(*l*_*j*_,*m*_*j*_),*j* = 0,…,3 be the control points of the *C*^2^ QTS curve $P(t)=\sum_{j=0}^{3}{d_{j}B}_{j}(t)$ for *t*∈[*t*_*i*_,*t*_*i*+1_]. Then, $P(t)=(x(t),y(t))=(\sum_{j=0}^{3}{l_{j}B}_{j}(t),\sum_{j=0}^{3}{m_{j}B}_{j}(t)).$

Now, $L(P(t))=\left(\begin{array}{c} a_{1}\sum_{j=0}^{3}{l_{j}B}_{j}(t)+a_{2}\sum_{j=0}^{3}{m_{j}B}_{j}(t)\\ +a_{3},b_{1}\sum_{j=0}^{3}{l_{j}B}_{j}(t),+b_{2}\sum_{j=0}^{3}{m_{j}B}_{j}(t)+b_{3} \end{array}\right).$ As we have $\sum_{j=0}^{3}B_{j}(t)=1\;for\;t\in [t_{i},t_{i+1}].$ Thus, $L(P(t))=(\begin{array}{c} a_{1}\sum_{j=0}^{3}l_{j}B_{j}(t)+a_{2}\sum_{j=0}^{3}m_{j}B_{j}(t)+a_{3}\sum_{j=0}^{3}B_{j}(t),\\ b_{1}\sum_{j=0}^{3}l_{j}B_{j}(t),+b_{2}\sum_{j=0}^{3}m_{j}B_{j}(t)+b_{3}\sum_{j=0}^{3}B_{j}(t) \end{array})=\sum_{j=0}^{3}(a_{1}l_{j}+a_{2}m_{j}+a_{3})B_{j}(t),\sum_{j=0}^{3}({b_{1}l}_{j}+b_{2}m_{j}+b_{3})B_{j}(t)=\sum_{j=0}^{3}(a_{1}l_{j}+a_{2}m_{j}+a_{3},{b_{1}l}_{j}+b_{2}m_{j}+b_{3})B_{j}(t)=\sum_{j=0}^{3}{{L(d}_{j})B}_{j}(t).$

**Fig 3:**
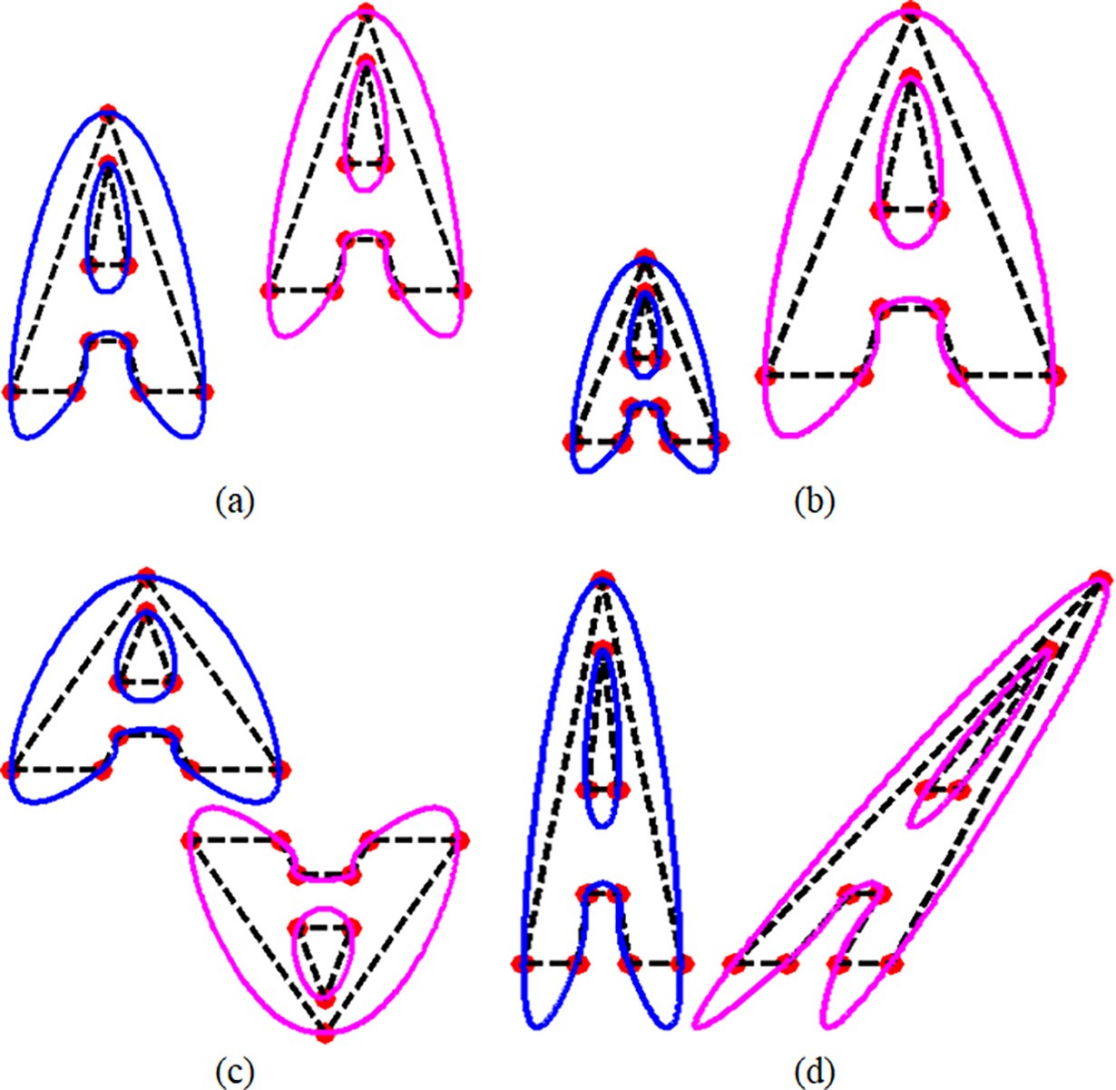
AIP using Data of Letter “A” and its corresponding QTS with (a) translation, (b) scaling, (c) rotation with angle *π* and (d) shearing.

The AIP proved in Preposition 2, is illustrated in Fig 3.

**Proposition 3 (Variation Diminishing Property (VDP)):** Consider the QTS curve for *t*∈[*t*_*i*_,*t*_*i*+1_], $P(t)=\sum_{j=0}^{3}{d_{j}B}_{j}(t)$ having control points $d_{j}=\{F_{i}{,V}_{i},W_{i},F_{i+1})\in R^{n}.$ Then any *N*−1 dimensional plane will intersect the QTS curve no more times than it will intersect the control polygon.

**Fig 4:**
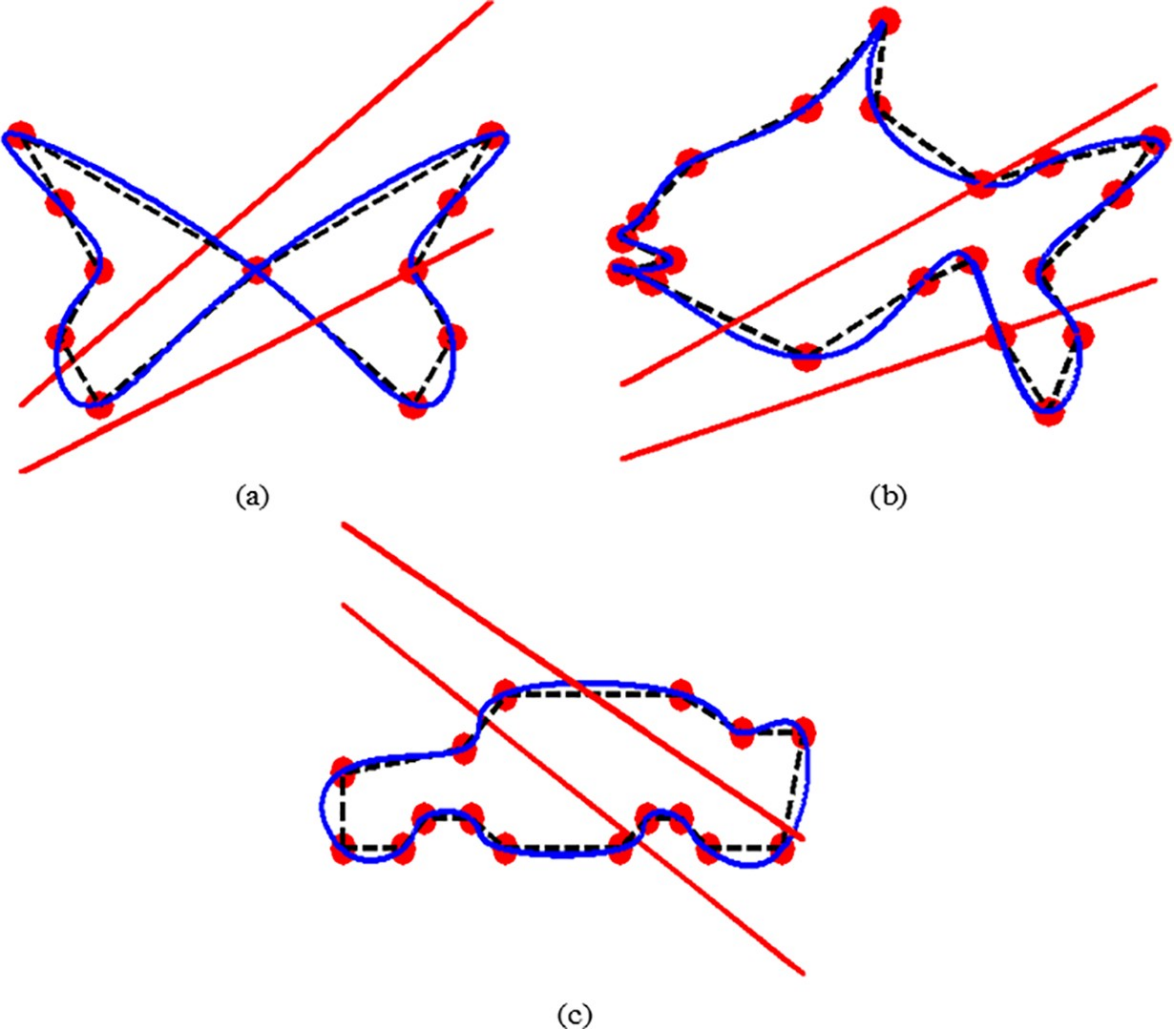
VDP for various object data and their corresponding QTSs: (a) Butterfly, (b) Fish, (c) Car.

The VDP is displayed, in Fig 4(A), (B) and (C), for different object data of Butterfly, Fish, and Car respectively. The interpolated QTS curves behave positively in all the model curves.

## 7. Comparison analysis

In this section, a brief comparison analysis of QTS and CPS is discussed. In this regard area of each spline, area between two spline curves QTS and CPS, visual differences of QTS and CPS, time elapsed of the two splines and error analysis of both splines are taken into account.

### 7.1. Area covered by CPS

The area of the CPS, defined in the interval [*t*_*i*_,*t*_*i*+1_], is derived as follows: $\int_{t_{i}}^{t_{i+1}}P_{i}(t)dt=\int_{t_{i}}^{t_{i+1}}\left[{\left(1-\theta \right)}^{3}F_{i}+{\left(1-\theta \right)}^{2}\theta V_{i}+(1-\theta )\theta^{2}W_{i}+(1-{\theta )}^{3}F_{i+1}\right]dt$. Here, $\theta =\theta (t)=(\frac{t-t_{i}}{h_{i}}),\;h_{i}=t_{i+1}-t_{i},0\leq \theta \leq 1$, and so, $d\theta =\frac{1}{h_{i}}dt$. Thus,

$$\int_{t_{i}}^{t_{i+1}}P_{i}(t)dt=h_{i}\int_{0}^{1}\left[{\left(1-\theta \right)}^{3}F_{i}+{\left(1-\theta \right)}^{2}\theta V_{i}+(1-\theta )\theta^{2}W_{i}+\theta^{3}F_{i+1}\right]d\theta =\frac{h_{i}}{4}(F_{i}+V_{i}+W_{i}+F_{i+1}),$$ (28)

### 7.2. Area covered by QTS

The QTS, defined by (1) in the interval [*t*_*i*_,*t*_*i*+1_], has the area to be derived as follows: $\int_{t_{i}}^{t_{i+1}}P_{i}(t)dt=\int_{t_{i}}^{t_{i+1}}\left[{\left(1-Sin\theta \right)}^{2}F_{i}+2(1-Sin\theta )Sin\theta V_{i}+2(1-Cos\theta )Cos\theta W_{i}+(1-Cos{\theta )}^{2}F_{i+1}\right]dt$, since, $\theta =\theta (t)=(\frac{t-t_{i}}{h_{i}})\frac{\pi }{2},\;h_{i}=t_{i+1}-t_{i}$, $0\leq \theta \leq \frac{\pi }{2}$, so, $d\theta =\frac{\pi }{2h_{i}}dt$. Thus,

$$\int_{t_{i}}^{t_{i+1}}P_{i}(t)dt=\frac{2h_{i}}{\pi }\int_{0}^{\frac{\pi }{2}}\left[{\left(1-Sin\theta \right)}^{2}F_{i}+2(1-Sin\theta )Sin\theta V_{i}+2(1-Cos\theta )Cos\theta W_{i}+(1-Cos{\theta )}^{2}F_{i+1}\right]\;d\theta =\frac{2h_{i}}{\pi }((\frac{3\pi }{4}-2)F_{i}+(2-\frac{\pi }{2})V_{i}+(2-\frac{\pi }{2})W_{i}+(\frac{3\pi }{4}-2)F_{i+1})=h_{i}((\frac{3}{2}-\frac{4}{\pi })F_{i}+(\frac{4}{\pi }-1)V_{i}+(\frac{4}{\pi }-1)W_{i}+(\frac{3}{2}-\frac{4}{\pi })F_{i+1})=h_{i}(.2267F_{i}+.2732V_{i}+2732W_{i}+.2267F_{i+1}).$$ (29)

### 7.3. Area analysis of QTS and CPS

It can be noticed from (28) and (29) that mathematically, the areas of the CPS and QTS are two different entities. This difference can also be noticed visually. For example, the QTS and the CPS are interpolated using the functions *f* = sin(*t*), tan(*t*) and *e*^*t*^. The area plots and the area between these two spline curves are demonstrated for area analysis of both splines.

**Fig 5:**
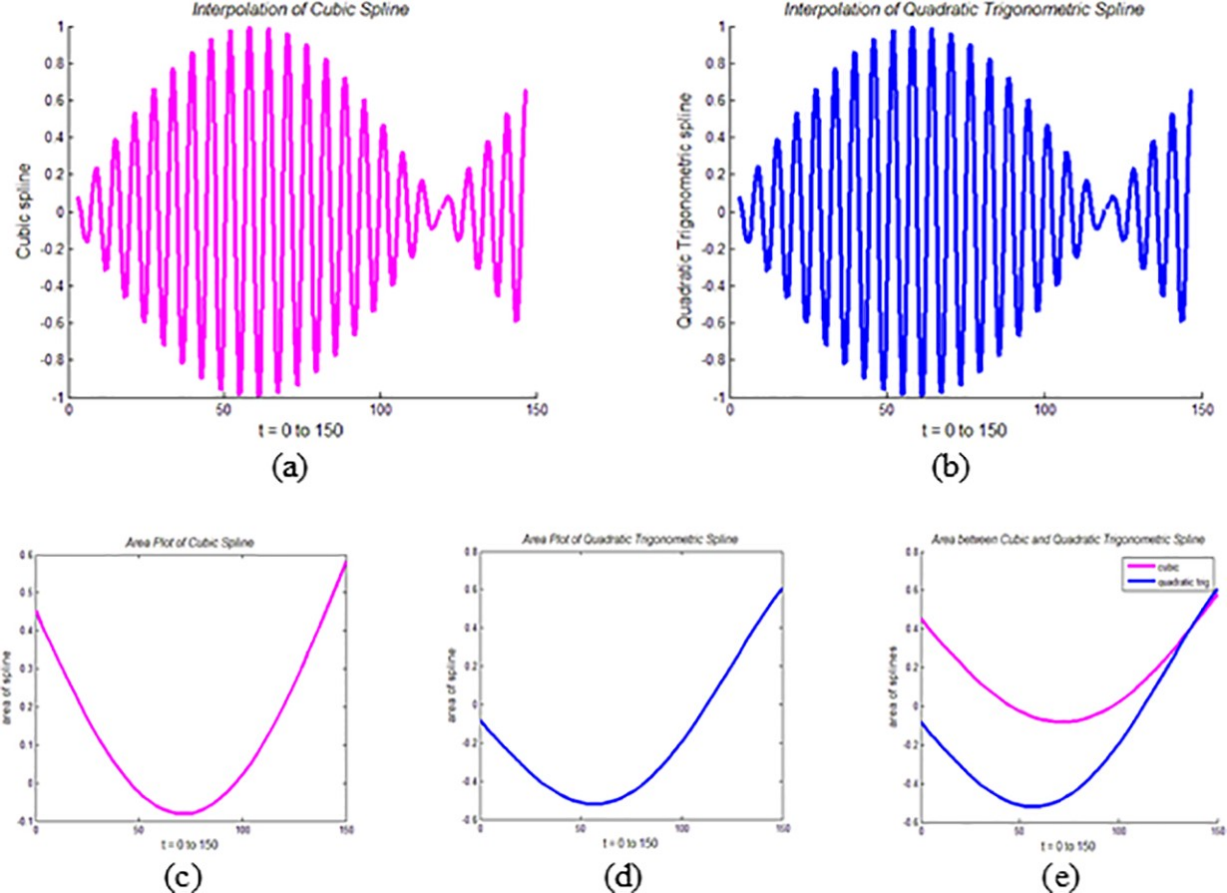
**Area Analysis of QTS and CPS using *f* = sin(*t*):** (a) Plot of CPS, (b) Plot of QTS, (c) Area plot of CPS, (d) Area plot of QTS, and (e) Area between CPS and QTS.

**Fig 6:**
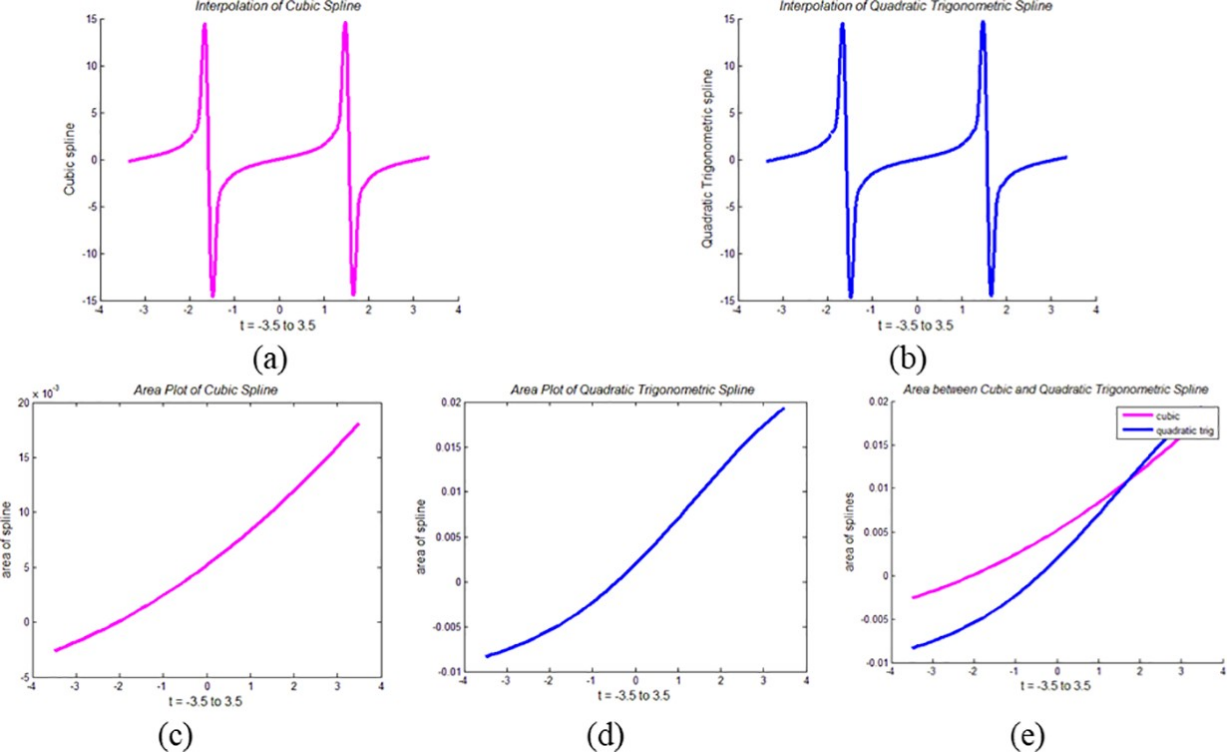
**Area Analysis of QTS and CPS using *f* = tan(*t*):** (a) Plot of CPS, (b) Plot of QTS, (c) Area plot of CPS, (d) Area plot of QTS, and (e) Area between CPS and QTS.

**Fig 7:**
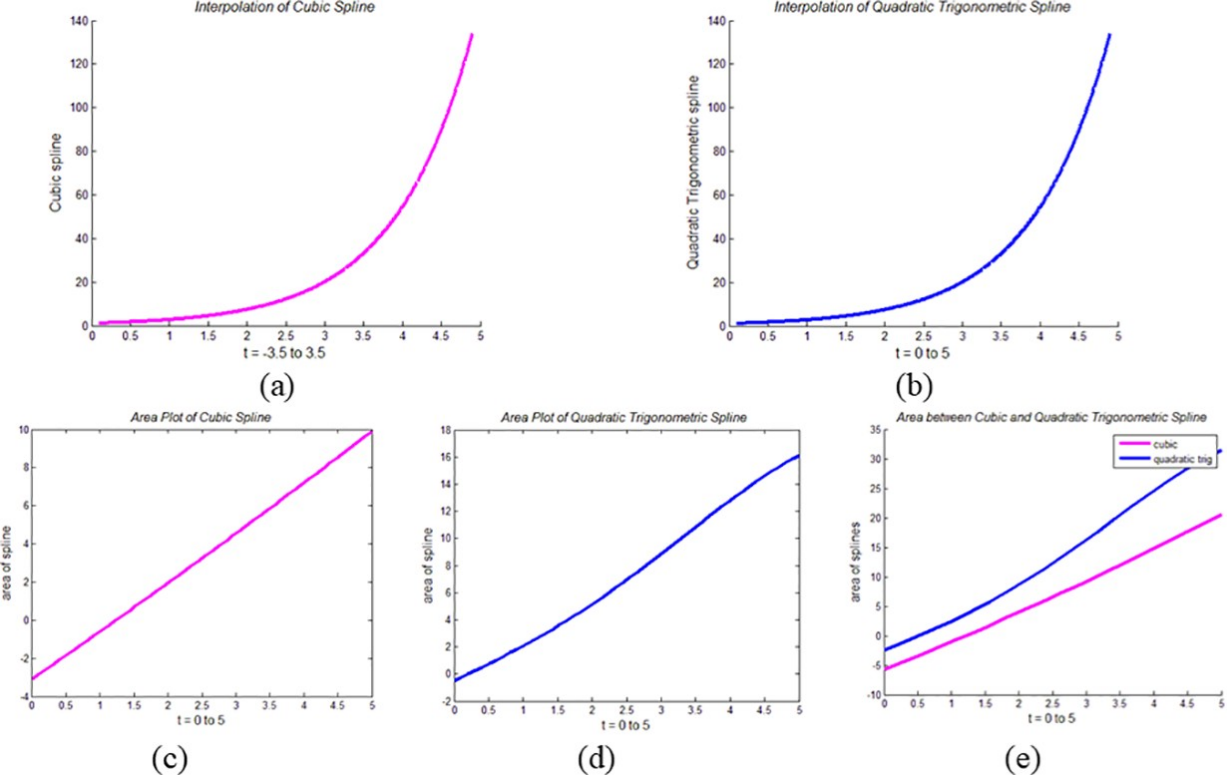
**Area Analysis of QTS and CPS using *f* = *e*^*t*^:** (a) Plot of CPS, (b) Plot of QTS, (c) Area plot of CPS, (d) Area plot of QTS, and (e) Area between CPS and QTS.

In Figs 5(A), 6(A) and 7(A), CPS interpolates the functions *f* = sin(*t*), tan(*t*) and *e*^*t*^, respectively, while in Figs 5(B), 6(B) and 7(B), these functions are interpolated by QTS. Figs 5(C), 6(C) and 7(C) demonstrate the area plots of the functions *f* = sin(*t*), tan(*t*) and *e*^*t*^, through the CPS, while Figs 5(D), 6(D) and 7(D) demonstrate the area plots of these functions by QTS. Finally, in the Figs 5(E), 6(E) and 7(E), area differences between two the splines CPS and QTS are demonstrated.

### 7.4. Visual difference between the CPS and QTS

The QTS interpolates almost like the CPS. However, because of different areas covered by them, their visual outputs are slightly variant from each other. To view the differences between the two splines CPS and QTS, the data of distinct existing objects is interpolated to produce design curves.

**Fig 8:**
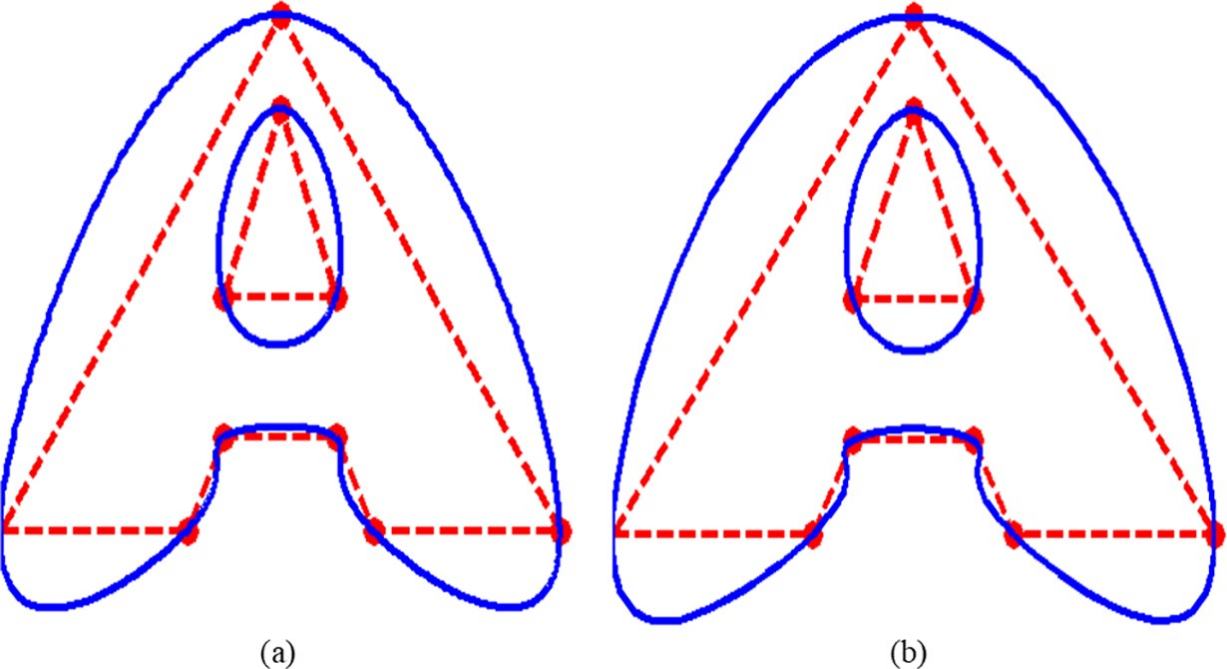
**Data of the letter “A” is interpolated:** (a) by CPS; (b) by QTS.

In Fig 8(A), data of the letter “A” is interpolated by CPS, while in Fig 8(B), the same object is interpolated using QTS. One can easily judge that the QTS is showing visually better smoothness than the CPS in almost every piece of curve. The curve in QTS is piece wisely more bulged outward as compared to its CPS counterpart.

**Fig 9:**
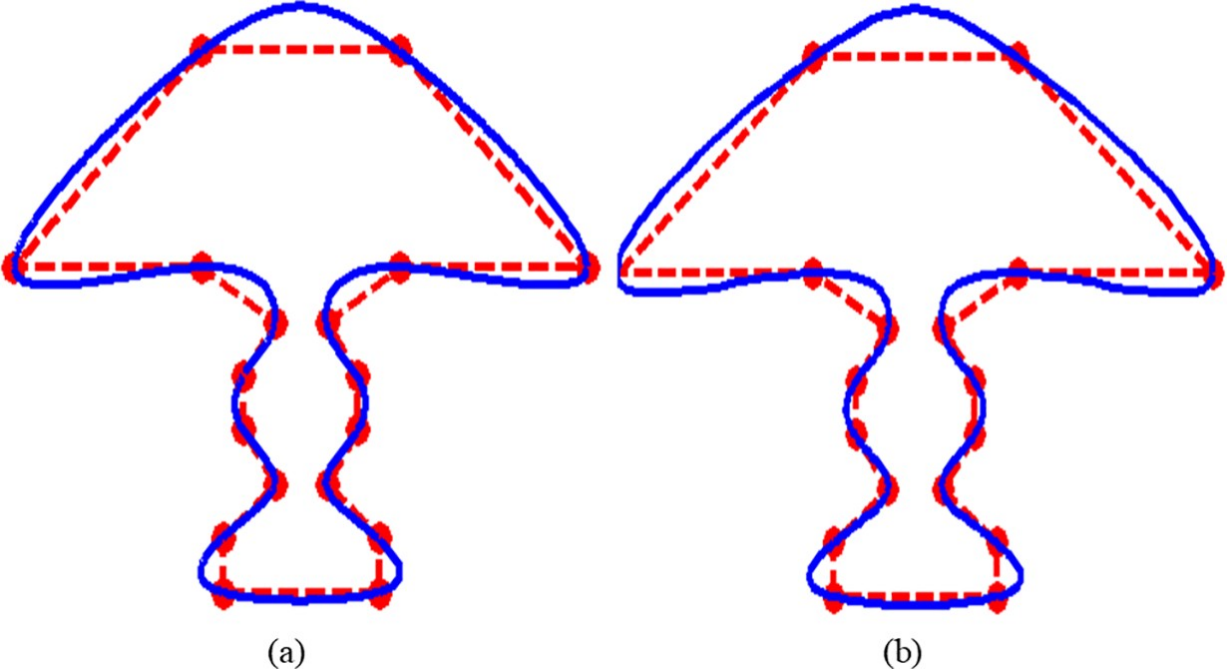
**Data of a lamp is interpolated:** (a) by CPS; (b) by QTS.

Like in Fig 8, one can notice similar to the behavior in Fig 9 too. In Fig 9(A), data of “Lamp” is interpolated by CPS, while in Fig 9(B), it is interpolated using QTS. It is very clearly judgable that the QTS shows visually better smoothness than the CPS in almost every piece of curve. The curve in QTS is piece wisely more bulged outward as compared to its CPS counterpart.

**Fig 10:**
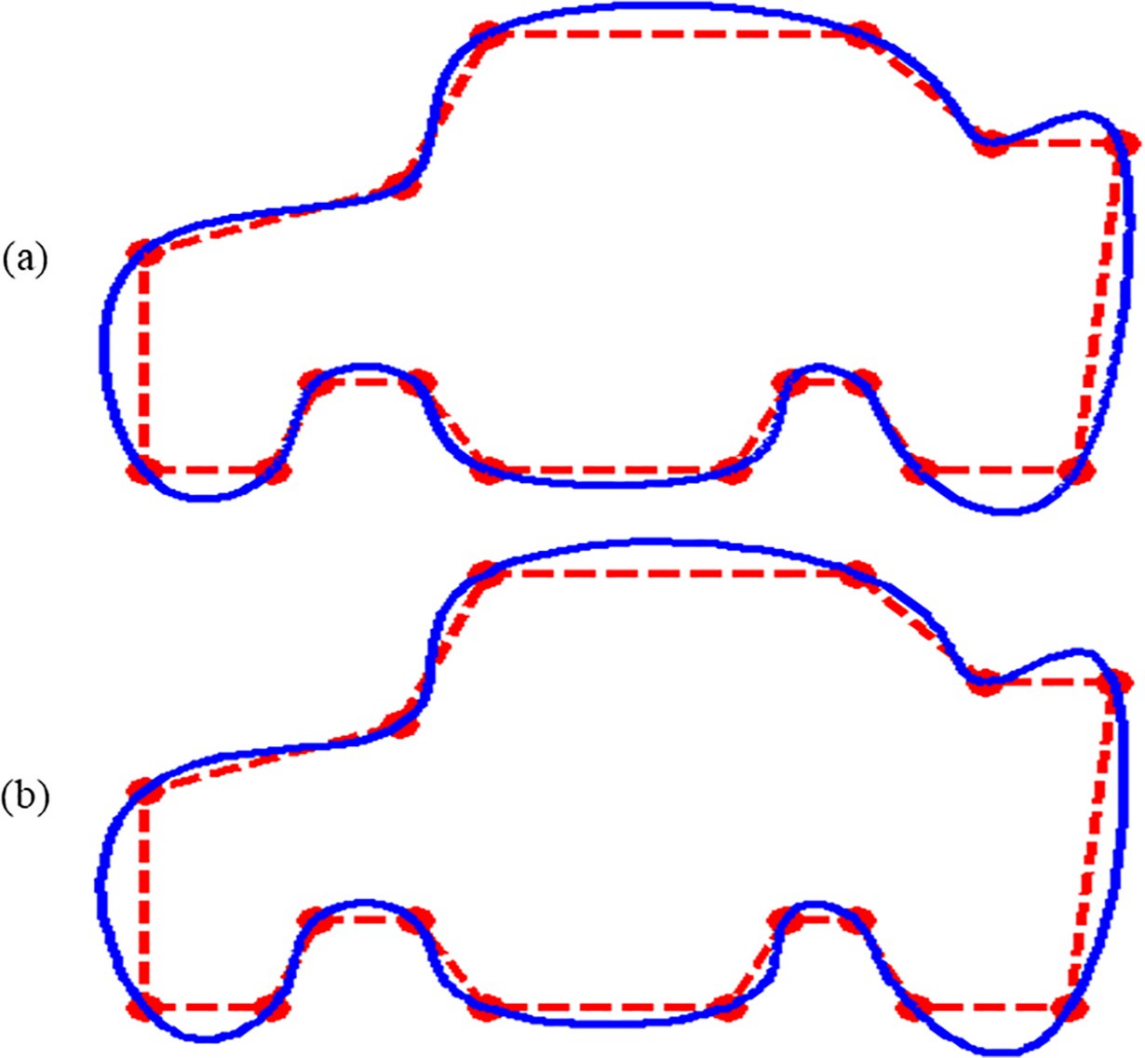
**Data of a car is interpolated:** (a) by CPS; (b) by QTS.

In Fig 10(A) and Fig 10(B), data of the “Car” is interpolated by CPS and QTS respectively. The QTS is showing visually smoother and bulging attitude in every piece of curve as compared to the CPS.

**Fig 11:**
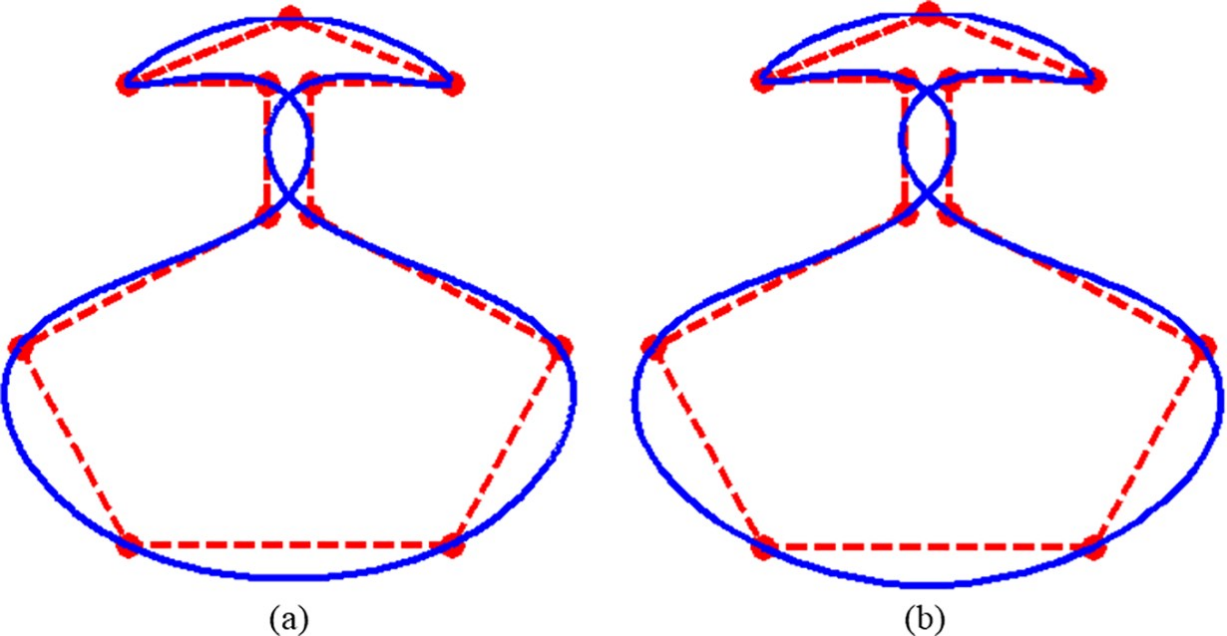
**Data of a vase is interpolated:** (a) by CPS; (b) by QTS.

Adding another example of a Vase in Fig 11, does not surprise to previous figures demonstrations. Fig 11(A) and Fig 11(B) are interpolated curves by CPS and QTS respectively. The QTS, as usual, is showing visually smoother and bulging attitude in every piece of curve as compared to the CPS.

### 7.5. Time elapsed by the two splines

The comparison analysis that the QTS is superior to CPS is also justified by comparing the time of execution of both the splines. Data of nine different objects, see Table 2, have been chosen for an extensive study.

**Table 2:** Table of time elapsed of both the splines.

#	Data Used	Time elapsed by CPS	Time elapsed by QTS	Difference between the times elapsed
		(in seconds)		
1	Fish	0.966504	0.328712	0.637792
2	Butterfly	0.494404	0.235974	0.258430
3	Car	0.877330	0.269906	0.607424
4	Lamp	0.850372	0.291029	0.559343
5	Guitar	1.024333	0.401127	0.623206
6	Letter ‘A’	0.561426	0.426110	0.135316
7	Flower	0.313687	0.230219	0.083468
8	Vase	0.435195	0.232384	0.202811
9	Ellipses	0.483795	0.245647	0.238148

In Table 2, different computed timings have been calculated for a variety of curves for data of nine objects mentioned in Column 2. The data in Column 3 represents the time elapsed by CPS, Column 4 represents the time elapsed by QTS, and Column 5 represents the difference of the times elapsed between CPS and QTS. It is very obvious to observe that the time elapsed by QTS is much smaller in quantity than that of the CPS. This difference is very significant as can be seen in Column 5 of Table 2. Hence, with a variety of experimentations, in Table 2, it is not difficult to decide that the proposed QTS is much faster curve interpolation scheme than the traditional CPS. Hence, QTS is a better substitute to CPS.

### 7.6. Error analysis for CPS and QTS

The error calculated by both CPS and QTS is defensible for comparing these splines. It is presented in Table 3 for six distinct trigonometric, logarithmic and polynomial functions *sin*(*t*),*cos*(*t*),*tan*(*t*),*sec*(*t*),*log*(*t*) and $\sqrt{t+6}+{\left(t+2\right)}^{2}$ shown in column 3. The domain of the functions are mentioned in column 2. Errors of these functions with CPS and QTS are shown in columns 4 and 5 respectively. Column 6 of Table 3 shows the difference of errors calculated by CPS and QTS. The difference, in column 6, between errors of both the splines is calculated to observe the accuracy of the splines.

**Table 3:** Table of error analysis, for CPS and QTS, for a variety of functions.

#	Value of *t* (taken)	Function used	Error calculated by CPS	Error calculated by QTS	Difference of Errors calculated by both splines
1	−*π*≤*t*≤*π*	*Sin*(*t*)	1.4439e-013	1.1924e-013	2.52E-14
2	−*π*≤*t*≤*π*	*Cos*(*t*)	1.2434e-014	1.2434e-014	0.000000
3	−*π*≤*t*≤*π*	*Tan*(*t*)	1.4550e-013	1.4530e-013	2.00E-16
4	−*π*≤*t*≤*π*	*Sec*(*t*)	1.2434e-014	1.2323e-014	1.11E-16
5	2≤*t*≤10	*Log*(*t*)	1.7764e-015	2.2204e-015	-4.44E-16
6	1≤*t*≤10	$\sqrt{t+6}+{\left(t+2\right)}^{2}$	4.2633e-012	4.2917e-012	-2.84E-14

## 8. Advantages of the proposed QTS

In this paper, a substantial method has been developed to construct a *C*^2^ QTS and a brief comparison analysis is discussed. The advantages of proposed scheme are comprehended as follows:

- The proposed QTS scheme has decent characteristics of trigonometric splines.
- The method keeps the suitable geometric properties of splines.
- It carries out the *C*^2^ smoothness.
- The proposed QTS produces an alternative to traditional CPS because of having four control points in its piecewise description.
- The comparison analysis of QTS and CPS, in Section 6, verifies the QTS as smother, more flexible, and more accurate alternate to CPS.
- The time analysis proves QTS computationally faster than CPS.

We can analyze the features of both QTS as well as CPS to see an overview of the two spline methods. It will highlight the differences between QTS and CPS. This is demonstrated in Table 4.

**Table 4:** Difference Table for QTS and CPS.

#	CPS	QTS
	CPS is algebraic piecewise polynomial.	QTS is trigonometric piecewise polynomial.
	CPS is Cubic.	QTS is Quadratic.
	CPS has four blending functions in an interval.	QTS has four blending functions in an interval.
	CPS owns the suitable geometric properties of splines.	QTS has the same geometric properties of splines as that of CPS.
	CPS has *C*^2^ continuity.	QTS has *C*^2^ continuity.
	The curve represented by CPS has reasonable bulge to represent flexibility.	The curve represented by QTS has more bulge than CPS.
	The area covered by the CPS is quite reasonable.	The area covered by the QTS is more than CPS.
	CPS is visually pleasing looking.	QTS is visually pleasing looking too and has more flexibility being able to have more bulge in the curve.
	CPS has reasonable computation error.	QTS observes less error of computation as compared to CPS and hence has more accuracy of the spline approximation.
	CPS elapses more time for execution.	The time elapsed by QTS is much smaller than CPS.
	CPS is economical in computation.	QTS is more economical in computation as compared to CPS.
	CPS is a well-known and recognized spline method used for Curve design.	QTS is a better alternative to CPS and hence expected to be more popular than CPS.

## 9. Conclusion

A *C*^2^ spline technique QTS is proposed and built with the eagerness of the object modeling using quadratic trigonometric functions. The curve model built through the proposed method owns the best suitable geometric properties such as partition of unity, CHP, AIP and VDP. The proposed scheme is more advantageous over the traditional CPS method. It is smoother, more flexible, faster and more accurate alternate to CPS. Furthermore, the built curve method is modest overall and is ideal for curve modeling. The authors, as future work, are also looking to expand the idea of the proposed QTS curve models for the designing of surface models.

## Supporting information

S1 Table Table of data of objects. (PDF)
