# Supplementary material for: A quadratic trigonometric spline for curve modeling
Source: PLoS One. 2019 Jan 10;14(1):e0208015. doi: 10.1371/journal.pone.0208015 (PMC6328157; doi:10.1371/journal.pone.0208015)
Supplement: S1 Table — (PDF) [file pone.0208015.s001.pdf]

#	Different Objects.		Data of the objects
(1)	Circle	*x*	0 1 2 1
		y	1 0 1 2
(2)	Lamp	*x*	0 0 .5 .2 .2 .5 -.2 -2 -.2 1.7 3.5 1.7 1 1.3 1.3 1 1.5 1.5
		y	0 .5 1 1.5 2 2.5 3 3 5 5 3 3 2.5 2 1.5 1 .5 0
(3)	Car	*x*	.5 1.4 1.7 2.4 2.9 4.6 5 5.5 5.9 7 7.3 6.4 5.5 2.9 2.3 0.5
		y	.5 .5 0.9 0.9 .5 .5 .9 .9 .5 .5 2 2 2.5 2.5 1.8 1.5
(4)	Flower	*x*	0 1 2 1 2 1 0 1
		y	0 1 0 1 2 1 2 1
(5)	Guitar	*x*	1.5 3 4 5.5 6 5 5.5 5 4 4 4 3 3 3 2 1.5 2 1
		y	1 .5 .5 1 3 4 5 6.2 7 9 12 12 9 7 6.2 5 4 3
(6)	Vase	*x*	2.5 1 2.3 2.3 0 1 4 5 2.7 2.7 4
		y	4 3.5 3.5 2.5 1.5 0 0 1.5 2.5 3.5 3.5
(7)	'A' alphabet	*x*	0 1 1.2 1.8 2 3 1.5
		y	0 0 1 1 0 0 5.5
